# Optimizing Lactic Acid Bacteria Proportions in Sourdough to Enhance Antifungal Activity and Quality of Partially and Fully Baked Bread

**DOI:** 10.3390/foods13152318

**Published:** 2024-07-23

**Authors:** Ricardo H. Hernández-Figueroa, Emma Mani-López, Nelly Ramírez-Corona, Aurelio López-Malo

**Affiliations:** Departamento de Ingeniería Química, Alimentos y Ambiental, Universidad de las Américas Puebla, San Andrés Cholula 72810, Mexico; ricardoh.hernandezf@udlap.mx (R.H.H.-F.); emma.mani@udlap.mx (E.M.-L.); nelly.ramirez@udlap.mx (N.R.-C.)

**Keywords:** sourdough, lactic acid bacteria, antifungal activity, bread quality, sensory evaluation, mixture design optimization

## Abstract

The organic acids produced by lactic acid bacteria (LAB) during the fermentation of sourdoughs have the ability to reduce the growth of different molds. However, this ability depends on the LAB used. For this reason, in this study, the proportions of different LAB were optimized to obtain aqueous extracts (AEs) from sourdough to reduce fungal growth in vitro, control the acetic acid concentration, and obtain a specific lactic to acetic acid ratio. In addition, the optimized mixtures were used to formulate partially baked bread (PBB) and evaluate the mold growth and bread quality during refrigerated storage. Using a simplex-lattice mixture design, various combinations of *Lactiplantibacillus plantarum*, *Lacticaseibacillus casei*, and *Lactobacillus acidophilus* were evaluated for their ability to produce organic acids and inhibit mold growth. The mixture containing only *Lpb. plantarum* significantly reduced the growth rates and extended the lag time of *Penicillium chrysogenum* and *P. corylophilum* compared with the control. The AEs’ pH values ranged from 3.50 to 3.04. Organic acid analysis revealed that using *Lpb. plantarum* yielded higher acetic acid concentrations than when using mixed LAB. This suggests that LAB-specific interactions significantly influence organic acid production during fermentation. The reduced radial growth rates and extended lag times for both molds compared to the control confirmed the antifungal properties of the AEs from the sourdoughs. Statistical analyses of the mixture design using polynomial models demonstrated a good fit for the analyzed responses. Two optimized LAB mixtures were identified that maximized mold lag time, targeted the desired acetic acid concentration, and balanced the lactic to acetic acid ratio. The addition of sourdough with optimized LAB mixtures to PBB resulted in a longer shelf life (21 days) and adequately maintained product quality characteristics during storage. PBB was subjected to complete baking and sensory evaluation. The overall acceptability was slightly higher in the control without sourdough (7.50), followed by bread formulated with the optimized sourdoughs (ranging from 6.78 to 7.10), but the difference was not statistically significant (*p* > 0.05). The sensory analysis results indicated that the optimization was used to successfully formulate a sourdough bread with a sensory profile closely resembling that of a nonsupplemented one. The designed LAB mixtures can effectively enhance sourdough bread’s antifungal properties and quality, providing a promising approach for extending bread shelf life while maintaining desirable sensory attributes.

## 1. Introduction

Sourdough is a natural preservation system that prolongs the mold-free shelf-life of bread [1,2,3]. Sourdough fermentation is a traditional bread-making process that uses lactic acid bacteria (LAB) and yeast to ferment the dough [3]. Numerous studies have reported that the antifungal activity of sourdough is mainly correlated with the presence of lactic and acetic acids [2,3,4,5,6,7,8,9]. This fermentation procedure is renowned for enhancing bread’s flavor, texture, and nutritional profile. One of the key advantages of sourdough fermentation is its ability to produce organic acids, such as lactic acid and acetic acid, which can inhibit the growth of spoilage microorganisms, including molds [2,7,8,10]. These bioactive compounds contribute to the bread’s sensory quality and extend its shelf life by preventing fungal growth [2,6,7,8,9,10].

The antifungal properties of sourdough are mainly influenced by the specific strains of LAB used during fermentation [2,7,8,10]. Different LAB produce varying amounts and types of organic acids and other antimicrobial compounds [7,8]. As a result, the effectiveness of sourdough in inhibiting mold growth can vary significantly depending on LAB activity. Numerous LAB strains have demonstrated significant antifungal activity, contributing to the inhibition of mold growth in sourdough bread. In addition to *Lactiplantibacillus plantarum* [2,7,11], *Lactobacillus acidophilus*, and *Lacticaseibacillus casei* [8], LAB such as *Levilactobacillus brevis*, *Limosilactobacillus reuteri*, *Limosilactobacillus fermentum*, *Fructilactobacillus sanfranciscensis* (formerly *Lactobacillus sanfranciscensis*), and *Pediococcus pentosaceus*, among many others, are also effective antifungals [4,12,13]. These LAB produce various bioactive compounds, including organic acids, bacteriocins, and antifungal peptides, which may inhibit fungal growth [2,14]. By utilizing LAB to produce antifungals, sourdough formulations can be enhanced to improve their quality, resulting in sourdough bread with good sensory acceptability and a longer shelf life [15].

Lactic and acetic acids play significant roles in creating sourdough bread’s distinct flavor and aroma. Sourdough bread benefits from the presence of lactic acid, which contributes to the unique flavor profile of the bread [16] and may affect the texture by enhancing the gluten network, which contributes to a softer crumb and better overall mouthfeel. Acetic acid is responsible for the sour taste of bread. Achieving the right balance between acetic and lactic acid is essential for a well-balanced flavor profile, as well as texture and overall acceptability, of sourdough bread [17,18,19].

Lactic and acetic acid concentrations and their molar ratio (quotient of fermentation (QF)) are important indicators used to describe sourdough’s features [20]. These organic acids are responsible for lowering the pH which, consequently, affects sourdough structure, sensory attributes, antifungal properties, and preservation [2,7,8,10]. The appropriate ratio of lactic acid to acetic acid typically ranges from 4:1 to 10:1. In practice, achieving this ratio involves controlling the fermentation conditions (time and temperature) and using a balanced starter culture [20,21], which may include homofermentative and heterofermentative LAB in appropriate proportions.

Understanding the balance of lactic and acetic acids in sourdough fermentation is crucial for enhancing the bread’s sensory attributes and inhibiting fungal growth on its surface. Achieving this balance involves managing the fermentation process to optimize their concentrations [22,23]. Both lactic and acetic acids lower the pH of the dough, creating an environment that is less favorable for the growth of spoilage microorganisms and fungi [12]. Due to its stronger antimicrobial properties, acetic acid is particularly effective against fungal growth. This balance can be managed through the selection of the starter culture. A balanced starter culture with homofermentative and heterofermentative LAB can help achieve the desired ratio, so selecting specific LAB known for their production profiles can be helpful.

Understanding and optimizing the balance of LAB in sourdough fermentation are essential for enhancing the bread’s sensory qualities and retarding mold growth. By taking advantage of mixture design approaches, this study aimed to provide valuable information on the optimal proportions of LAB that can maximize antifungal activity and improve the shelf life and sensory attributes of sourdough bread. LAB were selected based on their well-documented roles in fermenting sourdough bread [7,8]. *Lactiplantibacillus plantarum*, *Lacticaseibacillus casei*, and *Lactobacillus acidophilus* were chosen for their ability to produce organic acids during sourdough fermentation, which enhance flavor, texture, and shelf-life. A mixture of these LAB strains can maximize organic acid production and antifungal activity more effectively than a single strain, making them suitable for ensuring consistent sourdough bread production.

This study used a mixture design approach to determine the optimal combination of *Lpb. plantarum*, *L. acidophilus*, and *Lcb. casei* to identify the proportions that maximize organic acid production, maintain a desirable acetic/lactic acid balance, and effectively inhibit the growth of *Penicillium chrysogenum* and *P. corylophilum* in vitro by evaluating the aqueous extracts (AEs) of sourdough fermented with different LAB ratios. Furthermore, the impact of these optimized LAB mixtures on the quality and shelf life of partially and fully baked bread was assessed.

## 2. Materials and Methods

### 2.1. Culture Conditions

*Lactiplantibacillus plantarum* NRRL B-4496, *Lactobacillus acidophilus* NRRL B-4495, and *Lacticaseibacillus casei* 21/1 were obtained from the culture collection of the Food Microbiology Laboratory at UDLAP, cultured in de Man, Rogosa, and Sharpe (MRS) broth (Difco, BD, Sparks, MD, USA) at 35 °C for 48 h, and then collected via centrifugation [7]. *Penicillium chrysogenum* and *P. corylophilum* were obtained from the culture collection of the Food Microbiology Laboratory at Universidad de las Américas Puebla. The molds were grown on potato dextrose agar (PDA; Bioxon, BD, Ciudad de México, Mexico) slants for 7 days at 25 °C. The spores suspensions were obtained according to Hernández-Figueroa et al. [8] and adjusted to a concentration of ≈10^4^ spores/mL with Tween^®^ 80 solution (0.1%) (Sigma-Aldrich, St. Louis, MO, USA).

Sourdough (type II) was evaluated through three different essays: chemical analysis (from sourdough supernatants obtained after centrifugation and filtration), evaluation of antifungal activity in vitro (employing sourdough supernatant after centrifugation and heat treatment), and incorporating the sourdough into the bread formulation (partially and fully baked bread) to enable technological application.

### 2.2. Aqueous Extract Preparation

The AEs were obtained from a type II sourdough supplemented with *Lactiplantibacillus*, *Lacticaseibacillus*, or *Lactobacillus* following the methodology of Hernández-Figueroa et al. [7,8]. The proportions of LAB are shown in Table 1 according to the mixture design proposed in this paper. Each mixture was prepared with 200 g of wheat flour, 200 g of water, and a LAB weight/weight percentage according to the proportions obtained from the experimental design. The ingredients were mixed until a homogeneous dough was obtained, which was fermented at 35 °C for 48 h. The sourdoughs were centrifuged at 7000× *g* for 25 min at 5 °C (Sorvall ST 8R, Thermo Fisher Scientific, Osterode am Harz, Germany). The resulting supernatant was then filtered through a 0.45 μm cellulose nitrate filter (Advantec, MFS, Dublin, CA, USA) to obtain the AE. The AEs were then stored at −18 °C until they were analyzed [8].

### 2.3. Determination of pH, Titratable Acidity, and Organic Acids

The AEs were characterized by measuring pH using AACC method 02–52 with a calibrated pH meter (HI 2210 Hanna Instruments, Woonsocket, RI, USA). Titratable acidity (%TA) was determined using the AACC method 02–31 [24] and is expressed as lactic acid. Measurements were performed in triplicate.

The analysis and quantification of the lactic and acetic acid concentrations were carried out via high-performance liquid chromatography (HPLC) according to Hernández-Figueroa et al. [8]. An Agilent 1260 chromatograph (Agilent Technologies, Santa Clara, CA, USA) equipped with a diode-array detector (DAD) programmed to a wavelength of 210 nm was used, and the separation of compounds was performed with an Aminex HPX- 87H column (300 mm × 7.8 mm) (BIO-RAD, Hercules, CA, USA). Standard solutions (30–400 mM) of lactic and acetic acids were prepared and used as external standards to quantify the organic acids in the aqueous extracts. The peak area for each acid solution was correlated with the concentration using a linear model (R^2^ > 0.99). Each aqueous extract was injected, and, based on the retention times of the standards, the area under the curve was quantified to determine the concentration of each acid in the samples.

### 2.4. Radial Mold Growth Mathematical Modeling

The radial mold growth was evaluated according to Hernández-Figueroa et al. [8], with some modifications. The sourdoughs fermented for 48 h were centrifuged at 7000× *g* for 25 min at 5 °C, and the supernatant was thermally treated at 100 °C for 1 min (PAE). Sterile, melted PDA was mixed with the PAE at different concentrations (0, 25, or 50% *v*/*v*). Each PAE–agar was poured into Petri dishes (60 mm × 15 mm), allowed to solidify, inoculated with 5 μL of the spore suspension (≈10^4^ spores/dish) in the center of the plate, and incubated at 25 °C. The colony’s diameter was measured at three points every 24 h until the mold growth covered the entire diameter of the plate. The mold growth was modeled with the Baranyi equations (Equations (1) and (2)) [25] using the colony diameter evolution data obtained during incubation. The model allows estimating μ (maximum growth rate, 1/h), A (maximum growth, mm), and λ (the lag phase, h) via nonlinear regression.

To obtain the model parameters, the collected data were fitted by using DMFit (online application), proposed by Baranyi and Roberts [25] [Equations (1) and (2)], which is available for free (http://www.combase.cc, accessed on 7 June 2024). The models’ goodness of fit, residual analysis, and correlation coefficients (R^2^) were also calculated.

(1) $$D=\mu A-ln\left[1+\frac{\left[exp\left(\mu A\right)-1\right]}{exp\left(D_{max}-D_{0}\right)}\right]$$

(2) $$A=t+\left(\frac{1}{\mu }\right)ln\left[exp\left(-\mu t\right)+exp\left(-\mu t\right)-exp\left(-\mu t-\mu \lambda \right)\right]$$

*D* is the colony diameter (mm) at time *t* (h), *D*_0_ is the colony diameter at time 0, *D_max_* is the maximum growth diameter, *μ* is the maximum growth rate (1/h), *A*, (maximum growth diameter, mm) and *λ* is the phase of adaptation or lag time (h).

### 2.5. Experimental Design

A simplex-lattice mixture design (SLMD) was used to evaluate the effect of the three LAB (*Lpb. plantarum*, *X*_1_; *Lcb. casei*, *X*_2_; and *L. acidophilus*, *X*_3_) on the antifungal capacity of PAE, as well as the production of organic acids. Component proportions are expressed as fractions of the mixture, with a sum of one. Each factor level and experimental design are presented in Table 1. The 10 points evaluated included three single-component formulations, three two-component mixtures, and four three-ingredient mixtures. Two replicates of each point of the proposed experimental design were performed.

### 2.6. Modeling of Experimental Data

The polynomial equation (Equation (3)) in terms of the evaluated components (*x_i_*) was fitted for each analyzed response. This polynomial model differs from full polynomial models because it does not contain a constant term (intercept equal to zero). The polynomial model equation was

(3) $$y=\beta_{1}x_{1}+\beta_{2}x_{2}+\beta_{3}x_{3}+\beta_{12}x_{1}x_{2}+\beta_{13}x_{1}x_{3}+\beta_{23}x_{2}x_{3}+e$$

where *y* is the estimated response; *β*_1_, *β*_2_, *β*_3_, *β*_12_, *β*_13_, and *β*_23_ are constant coefficients for each linear and nonlinear (interaction) term obtained for the prediction models of the processing components. Minitab 20 statistical software (Minitab LLC, State College, PA, USA) was used to estimate the polynomial equations. The fitted models were subjected to analysis of variance (ANOVA), and we calculated the determination coefficient (R^2^) and lack of fit. After removing the nonsignificant terms (*p* > 0.05), the best-fit equations for responses (pH, TA, lactic and acetic acid concentrations, ratio lactic/acetic, and mold growth parameters) were established. Multiple response optimizations were conducted to recognize the combination of experimental factors that simultaneously optimized the selected responses.

Based on the predicted polynomial equations, the lag time, the concentration of acetic acid (this acid has been reported [8] to be an important antifungal agent), and the ratio of lactic acid to acetic acid was optimized. The aim was to maximize the lag time for both molds; for the concentration of acetic acid, a range of 150–170 mM with a target of 160 mM was used; and for the logarithm of the ratio of organic acids, which ranges between −0.67 and 1.97, 0.65 was selected as the target, since a ratio between 0.5 and 1.0 is more acceptable for bread flavor [26]. Two optimizations were performed by considering two different sets of weighting factors. In the first one, the assigned weights were 0.50 to the ratio of organic acids, 0.15 to the lag time of each tested mold, and 0.20 to the concentration of acetic acid. The second optimization involved assigned weights of 0.50 to the ratio of organic acids and 0.25 to the lag time of each tested mold.

Typically, the optimized conditions should be tested in vitro for antifungal activity. However, the technological application of sourdough is expected to be made into bread. Therefore, this study tested the optimal mixtures by incorporating sourdough as an ingredient in the bread formulation to evaluate the antifungal activity and the impact on bread quality.

### 2.7. Partially and Completely Baked Bread Preparations

A standard formulation of white bread was used to prepare the samples: 38% wheat flour, 23% water, 1% yeast, and 38% type II sourdough [8] formulated with the proportions of the two optimized mixtures. The dough was mixed and kneaded using a Legacy mixer/kneader (Hobart, Troy, OH, USA). The dough was then divided into pieces weighing 60 g each. These pieces were allowed to ferment for 60 min at 30 °C in a Mini combo oven (Zucchelli Alpha, Trevenzuolo, Verona, Italy). After fermentation, the dough was partially baked at 200 °C for 15 min in a Mini combo electric oven (Zucchelli Alpha, Trevenzuolo, Verona, Italy). The partially baked bread was then cooled at 25 °C for 1 h. The partially baked bread (PBB) was packaged into polyethylene pouches and stored in a refrigerator at 4 ± 1 °C [27]. Based on previous reports [28], we analyzed the partially baked bread pieces that had been refrigerated for up to 28 days. The PBB was completely baked at 200 °C for 10 min, then fully baked bread (FBB) was cooled at 25 °C for one hour.

### 2.8. Determination of Physicochemical and Quality Properties of Bread

pH measurements of PBB and FBB were conducted using a pH meter, model HI2210 (Hanna Instruments, Woonsocket, RI, USA), following AACC method 02-52 [24]. The pH was measured at 0, 7, 14, 21, and 28 days of storage. The crumb and crust from the FBB loaves were separated for the moisture and a_w_ tests, and they were analyzed individually at 0, 7, 14, 21, and 28 days of storage. The moisture content was determined using the AOAC 930.15 method [7], and the a_w_ analysis was performed with AquaLab Series equipment (Meter Food, Pullman, WA, USA). All measurements were carried out on the whole bread, and specific volume were determined according to AACC method 10–05 [24]. The width/height ratio was measured [29] using an electronic Vernier caliper. All measurements were conducted in triplicate.

The total titratable acidity in the PBB was determined using AACC method 02–31 [24]. The analysis and quantification of lactic and acetic acid concentrations were carried out via high-performance liquid chromatography (HPLC): both measurements were taken at the beginning and at the end of storage when fungal growth on the bread was visible. The hardness of the PBB and FBB was measured with an EZ-SX texture analyzer (Shimadzu Corporation, Kyoto, Japan) on a 2.5 cm thick slice of bread compressed (50%) with a stainless steel cylinder probe (d = 25 mm) at 60 mm/min speed [27]; the maximum force (peak) was registered as the force (N) of the curve analyzed using texturemeter software (Trapezium X, Shimadzu Corporation, Kyoto, Japan). The hardness was measured of both types of bread (PBB and FBB) at 0, 7, 14, 21, and 28 days of storage. All determinations were performed in triplicate.

### 2.9. Sensory Analysis

A total of 30 untrained judges were invited to evaluate the sensory quality of the fully baked bread loaves (PBB was stored at 4 °C for 24 h prior to baking), including bread without sourdough and bread with type II sourdough supplemented with the optimized mixtures. Seven attributes of bread (odor, color, crumb appearance, crust appearance, flavor, texture, and overall acceptability) were evaluated using a 9-point hedonic scale [8]. Participants in the sensory evaluation tests provided informed consent by acknowledging the following statement: “I understand that my responses are confidential, and I consent to taking part in this sensory evaluation”. Only those who agreed to this statement were allowed to participate. They were also informed that they could withdraw from the test at any time without providing a reason. We also explicitly stated, “The products being tested are safe for consumption”. We assured participants that their data would not be disclosed without their knowledge.

### 2.10. Storage for Visual Examination of Mold Growth

The packaged partially baked bread loaves stored at 4 ± 1 °C were observed daily (for 28 days) or until visible mold growth was detected, looking for any signs of mold growth, such as fuzzy or slimy spots of white, green, blue, or black colors, and looking for unusual discoloration of the bread crust. These observations allowed the testing the antifungal effectiveness of the optimized sourdoughs [7,8].

### 2.11. Statistical Analysis

The results are shown as mean values and standard deviations. The data were studied using analysis of variance (ANOVA). Pairwise comparisons for mean values were conducted using Tukey’s test at a significance level of *p* < 0.05. The statistical software Minitab 20 (Minitab LLC, State College, PA, USA) was used for the analysis.

## 3. Results and Discussion

### 3.1. Modeling Mold Radial Growth Inhibition

**Figure 1:**
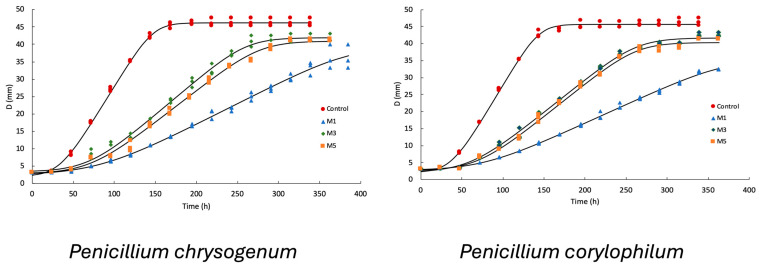
Radial mold growth and Baranyi equation prediction from the lactic acid bacteria mixtures obtained from the simplex-lattice mixture design (SLMD). Solid lines indicate the model prediction; M1, M3 and M5 correspond to different LAB mixtures (Table 1).

The Baranyi prediction models had a good fit (R^2^ > 0.98). Figure 1 shows the radial growth graph of both molds under regular conditions (control) and when three of the mixtures of LAB with the greatest antifungal activity (M1, M3, and M5) were added. As can be seen, the growth rate significantly decreased (*p* < 0.05) in both molds compared with the control, which indicated the capability of the LAB to produce bioactive compounds with fungistatic activity such as organic acids. In particular, the mixture that contained only *Lpb. plantarum* (M1) presented a greater capability to reduce the growth rate of both molds. The evaluated mixtures significantly increased (*p* < 0.05) the lag time for both molds compared with that of the control.

### 3.2. Physicochemical Characteristics and Antifungal Activity of the Aqueous Extracts

Table 2 shows the pH values, titratable acidity, and concentration of organic acids (lactic and acetic acids) of the AEs of the sourdoughs fermented with the different mixtures of LAB. As shown, the pH of all the mixtures ranged from 3.50 to 3.04, with mixtures 1 (*X*_1_ = 1; *X*_2_ = 0; *X*_3_ = 0) and 9 (*X*_1_ = 0; *X*_2_ = 0.5; *X*_3_ = 0.5) having the lowest pH. In both mixtures, the proportion of *Lpb. plantarum* was the largest. The pH of the AEs agrees with previous reports which were between 3.10 and 4.10 depending on the LAB and the type of fermentation (homofermentative or heterofermentative) [6,7,8,28,29,30].

The organic acid (lactic acid and acetic acid) concentrations in the AEs depended on the LAB type and their interactions during fermentation. For instance, mixture 1, which only contained *Lpb. plantarum*, had the highest acetic acid concentration (≈560 mM) and a relatively high lactic acid concentration (≈120 mM); however, when *Lpb. plantarum* was added in equal parts (0.5) with the other LAB (*Lcb. casei* or *L. acidophilus*), the final concentration of acetic acid was significantly (*p* < 0.05) lower (≈6 mM) and that of lactic acid was more than double (≈270 mM).

The metabolic activity and behavior of LAB in sourdough fermentations are significantly affected by acidic environments, the production of antimicrobial compounds (organic acids) from other microorganisms, and carbohydrate metabolism in producing energy [31,32]. In general, the wheat flour fermentations used to produce sourdoughs have optimum conditions for heterofermentative LAB growth [32]. Therefore, it was expected that most of the mixtures analyzed in this study would produce lactic acid and acetic acid. Mixture 7, formulated with *L. acidophilus*, produced more lactic acid than acetic acid because *L. acidophilus* is an obligate homofermentative. Mixture 5 also produced a low amount of acetic acid, and this may have been because the proportion of *L. acidophilus* was higher than that of the heterofermentative LAB (*Lcb. casei* and *Lpb. plantarum*). Similarly, when equal parts of the three LAB were used (0.33) in mixture 6, the production of acetic acid was low; this may have been due to competition for nutrients and survival adaptability between the LAB.

The PAEs’ fungistatic effects are presented in Table 2. The radial growth rate was decreased in every tested mixture compared with that of the control, being 0.38 (1/h) and 0.40 (1/h) for *P. chrysogenum* and *P. corylophilum,* respectively. Also, the lag phase (Table 2) was longer with all tested mixtures compared with that the control, which was 33.79 (h) and 37.31 (h) for *P. chrysogenum* and *P. corylophilum,* respectively. These results demonstrated the PAEs’ fungistatic activity of sourdough fermentations with the LAB mixtures. The fungistatic activity of AEs and sourdoughs has been reported by other authors: they reduce the growth rate of different *Penicillium* species primarily because of their organic acids and, in some cases, peptide compounds [7,8,33,34,35,36]. Likewise, adding sourdough to the bread formulation almost doubles the product’s shelf-life [34].

The analyzed variables (Table 2) were subjected to statistical evaluation using the polynomial model associated with the mixture design solution. The effects of the LAB mixtures on the pH, TA (%), concentration of organic acids, mold growth response (rate and lag time), and proportion of lactic and acetic acids were evaluated. Table 3 presents the model coefficients obtained for each response. A lactic to acetic acid ratio of between 4 and 5 improves the sensory properties of bread [26]. Because of the wide range of the lactic/acetic ratio (0.2–93), the ratio was computed in log base (log R), resizing these ratio values to the range of −0.6 to 1.97, improving the model’s fit and enabling the optimization process to be carried out. Table 3 includes the correlation coefficient (*r*) and the results of the lack-of-fit test, indicating that the models appropriately explained the variability in the analyzed responses and that no significant differences (*p* > 0.05) existed between the observed and predicted values, respectively.

**Figure 2:**
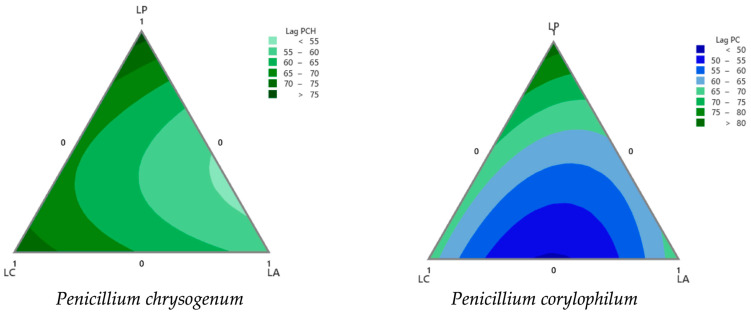
Contour plots were obtained from a simplex-lattice mixture design (SLMD) for different lag time (h) values for each tested mold. LP: *Lactiplantibacillus plantarum* NRRL B-4496; LC: *Lacticaseibacillus casei* 21/1, and LA: *Lactobacillus acidophilus* NRRL B-4495.

The contour plots (Figure 2) illustrate how *Lpb. plantarum* (LP), *Lcb. casei* (LC), and *L. acidophilus* (LA) proportions affected the lag times of the molds (*P. chrysogenum* and *P. corylophilum*). For *P. corylophilum*, the center of the triangle represents shorter lag times. Lag time increased with increasing proportions of LP. The LC and LA vertices indicated LC and LA had shorter lag times than LP, suggesting that higher proportions of LC and LA favored shorter lag times. For *P. chrysogenum*, as the proportion of LP increased, the lag time also increased. The LC vertex also shows moderate but shorter lag times than the LA vertex. This suggested that higher proportions of LA significantly reduced the lag time for *P. chrysogenum*, whereas higher proportions of LP increased it. Both molds showed increased lag times for higher LP proportions, but the effect was more pronounced for *P. corylophilum*. *P. chrysogenum* was particularly tolerant to LA proportions, achieving the shortest lag time with its derived sourdough. For *P. corylophilum,* with a lag between 75 and 80 h, the mixture had a higher proportion of *Lpb. plantarum* (>0.8) and low proportions of both *Lcb. casei* and *L. acidophilus* (<0.2) (Figure 2). The same behavior was observed for the lag time of *P. chrysogenum*.

**Figure 3:**
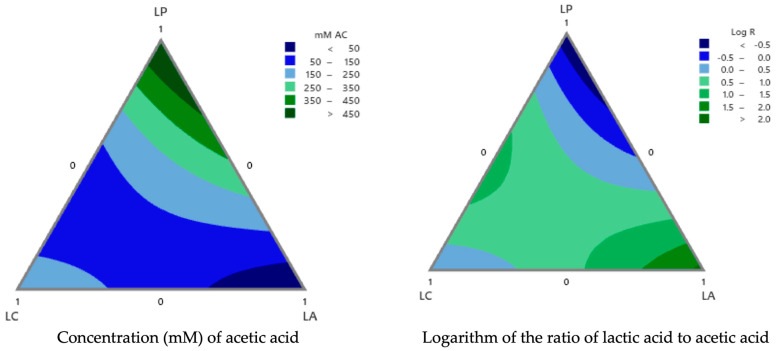
Contour plot of the concentration (mM) of acetic acid and logarithm of the ratio of lactic acid to acetic acid (log R) across different mixture proportions. LP: *Lactiplantibacillus plantarum* NRRL B-4496; LC: *Lacticaseibacillus casei* 21/1, and LA: *Lactobacillus acidophilus* NRRL B-4495.

Figure 3 presents contour plots of the acetic acid concentration expressed in mM and the logarithm of the relationship between the lactic acid and acetic acid concentrations (log R) across different mixture proportions. Figure 2 and Figure 3 were used to optimize the responses (growth lag time for molds *P. corylophilum* and *P. chrysogenum*, balanced log R value, and acetic acid concentration). Higher acetic acid concentrations were observed towards the upper regions of the contour plot where LP was dominant. To obtain high acetic acid concentrations (350–440 mM; Figure 3), since it has important antifungal activity, the mixtures consisted of a greater portion of *Lpb. plantarum* and small proportions of *Lcb. casei* and *L. acidophilus* (Figure 3). These results agree with those in the literature, which reports the ability of *Lpb. plantarum* to produce organic acids (lactic and acetic acids) and peptide compounds with antimicrobial activity. Both bioactive compounds present synergetic interactions when used as antifungal agents [2,7,37]. An appropriate ratio of lactic to acetic acid, which can enhance the sensory attributes of bread, typically falls between 4:1 and 10:1 [20], corresponding to a log R range of 0.5 to 1.0. Figure 3 illustrates that these log R values could be achieved using different mixtures of lactic acid bacteria, indicating that by adjusting the proportions of these components, the acceptability of bread within the specified log R range could be optimized.

From the analysis of the polynomial models, two optimizations (Table 4) were obtained, considering maximizing the lag time of both molds and searching for target values for the acetic acid concentration (160 mM) and the log R (0.65). The contour plots (Figure 2 and Figure 3) were analyzed to optimize the proportions of LP, LC, and LA in the mixture and achieve the desired outcomes. Higher acetic acid concentrations were obtained toward the upper regions controlled by LP. However, the central region, where all three components were balanced, was optimal for achieving around 160 mM acetic acid. The area for the log R value close to the target (0.65) was achieved with a balanced proportion of LP, LC, and LA.

When optimizing multiobjective mixture designs, assigning different response weights prioritizes specific goals. This approach considers the differences in the importance of the selected responses in achieving the desired outcome [38,39]. The objective function is a weighted sum, with all weighing factors being positive and summing up to one. These weights indicate the relative importance of each response in the process and help with prioritizing critical responses, balancing competing goals, and managing trade-offs. In the case analyzed, log R (target: 0.65) was assigned a weight of 0.50, reflecting high importance. The lag times of *P. corylophilum* and *P. chrysogenum* (both targeted to maximize) were assigned weights of 0.25 each, indicating that they were equally important but secondary to log R (Table 4). This helped to maximize the composite desirability function. In the other case, the acetic acid concentration (mM) was also included, and the assigned weights are shown in Table 4. This strategic weighting ensured that, although all responses were optimized, log R was emphasized due to its higher weight, leading to a well-balanced and effective solution, where a greater weight was given to the sensory part given the application of this type of sourdough. Selecting the correct optimization targets is essential when designing a sourdough bread mixture for improved shelf-life and sensory acceptability. In this case, the lag time for mold growth, the log R, and the acetic acid concentration were specifically important. Mold growth is a common spoilage issue in bread, and controlling it is essential for maintaining product quality and safety. Mold lag time can be considered a direct indicator of a bread’s shelf-life [14,40]. The ratio of lactic to acetic acid (log R) affects sourdough bread’s sensory attributes. The balance between these acids is thus fundamental for achieving the desired flavor profile [38,41]. Since different LAB (homofermentative and heterofermentative) produce varying amounts of lactic and acetic acids during sourdough fermentation, optimizing their proportion helps with controlling the overall fermentation process and end-product quality. While acetic acid is essential for its preservative effect [21], since it helps extend the bread’s shelf-life by inhibiting spoilage organisms’ growth, including molds [39], its concentration must be optimized to prevent the bread from having a sour taste.

Table 5 shows the optimal LAB mixture proportions found from the statistical analysis. For optimization 1 (OP1), the composite desirability of 0.925 reflected the optimization’s overall success in balancing all three responses. A desirability close to one indicates effective optimization among different goals. The optimization yielded a mixture composition that nearly achieved the target for log R and significantly maximizes lag time for both studied molds within their acceptable ranges.

For optimization 2 (OP2), the optimal mixture composition of *Lpb. plantarum*, *Lcb. casei,* and *L. acidophilus* produced high desirability scores for all responses, indicating that the chosen mixture was highly effective in achieving the set goals. The composite desirability (0.870) reflected a well-optimized overall solution. The three-component mixture design optimized the three selected responses appropriately, meeting the overall criterion (desirability function > 0.85). The correlation coefficients and lack-of-fit tests indicated a reliable explanation of the response variability and accurately predicted values.

### 3.3. Bread Quality and Physicochemical Properties

Sourdough was formulated according to the concentrations indicated in the two optimizations for bread testing. Table 6 shows the results of the PBB’s quality properties and physicochemical characteristics. The specific volume and the width-to-height ratio of all the samples were similar (*p* > 0.05); however, bread to which sourdough was added had slightly smaller values for both parameters than the control. Similar results regarding specific volume and width/height ratio have been reported by other authors [8,42,43], and it has been described that these quality factors are directly related to the adequate formation of gluten. Nutter et al. [44] observed the fermentation of LAB in dough, which modified the gluten microstructure and caused protein depolymerization. Similarly, Hernández-Figueroa et al. [8] and Minervini et al. [45] observed that when the pH was < 4, the organic acids produced by LAB could be found in undissociated form, which could cross the cytoplasmatic membrane of yeast and retard their growth, especially that of *Saccharomyces cerevisiae*. As shelf-time increases, the pH decreases; therefore, the TA increases for all breads. This indicates the survival of LAB in the PBB and postacidification activity. The control bread had significantly higher pH values (6.54 and 5.83; *p* < 0.05) and lower TA (0.2–0.4%; *p* < 0.05) than those of the pieces of bread to which sourdough had been added. Similar pH values were found by Zhao et al. [46] and Hernandez-Figueroa et al. [8] for loaves of bread made only with wheat flour, without sourdough. The bread formulated with sourdough from the LAB mixture under optimization 2 had the lowest pH (4.38 and 4.19; *p* < 0.05) and the highest TA (0.80–0.95%; *p* < 0.05). Various studies have shown that bread with a pH between 4.0 and 3.72 can be produced by adding sourdough fermented with different LABs, such as *Lpb. plantarum*, *Lactobacillus rossiae*, *Lactobacillus sakei*, and *Lactobacillus sanfranciscencis* [5,7,8,29,47].

Gunduz et al. [48] found that rapid acidification is essential for quickly performing sourdough fermentation. The authors mentioned that starter culture inoculation resulted in faster acidification than that with uninoculated control sourdough; thus, this intentional inoculation may ensure the reproducibility and stability of sourdough production. Also, the authors mentioned that LAB or strain selection is a promising strategy for developing starter cultures for controlled fermentations. Gunduz et al. [48] concluded that *Lpb. plantarum* and *Fructilactobacillus sanfranciscensis* could be applied as dual starter cultures in industrial sourdough production to reach the desired levels of acidification and aroma in a short fermentation processes. Gunduz et al. [48] concluded that developing starter culture combinations can be a successful strategy for several applications of sourdough fermentation.

Similarly, Gül et al. [26] investigated the use of three different LAB (*Lactobacillus curvatus* N19, *Weissella cibaria* N9, and *L. brevis* ED25) as type II sourdough culture starters to develop sourdough bread. The concentrations of lactic (23.54–31.86 mM) and acetic (7.16–12.83 mM) acids in the sourdough bread were significantly higher than those in the control (fermented only with commercial yeast) (3.11 and 1.50 mM, respectively). The authors found that a lactic to acetic acid ratio of the bread of between 2 and 2.7, known as the fermentation rate, was linked to better recognition of sourdough bread by consumers. They obtained lactic/acetic ratios in the range of 3.73 to 5.61, which are close to the recommended values for bread made with sourdough containing *L. curvatus* N19, resulting in an adequate sensory balance. Despite being similar to other sourdough loaves, these ratios are higher due to a lower amount of acetic acid.

The a_w_ (Table 6) of the PBB did not significantly change with the addition of sourdough or storage time (*p* < 0.05). All bread pieces presented the same a_w_ values (0.93) at the beginning of storage. After 18 days, the control bread presented a slightly higher a_w_ (0.95); for the bread with added sourdough, the a_w_ values after 28 days were 0.94 and 0.95 for optimization 1 and 2, respectively.

The PBB’s moisture content is presented in Table 6; the storage time does not significantly affect (*p* > 0.05) the moisture content. For the control bread, the moisture was 40.68 and 40.07% at the beginning and end of storage, and these values were higher compared with those of the bread pieces containing sourdough, which had initial and final values of 33.93 and 33.50% for the bread from optimized mixture 1 and 37.66 and 37.67% for bread from optimized mixture 2. The water retention in baking products depends primarily on the correct formation of the gluten network and starch granules. The formation of the gluten network and the swelling of starch granules reduce the loss of water vapor during the baking process [8,12,49]. Lactic acid bacteria can impact the gluten network primarily by depolymerizing proteins. Studies have shown that LAB release amylases, leading to decreased pH and the activation of wheat proteases. As a result, starch granules and gluten networks are affected, leading to reduced water vapor retention during baking. These structural changes could have been responsible for the lower moisture content of the bread that was supplemented with sourdough.

Table 7 shows the quality parameters and physicochemical characteristics of the FBB. In general, the moisture content of the bread containing sourdough was lower in both the crust and crumb (20.58 and 40.32% for the bread from optimized mixture 1; 26.76 and 42.07% for the bread from optimized mixture 2) with respect to the control (28.10 and 46.52%). The a_w_ of the crust for all samples ranged from 0.84 to 0.85 and did not show significant differences among them (*p* > 0.05). The crumb’s a_w_ was higher than that in the crust in all samples in the range of 0.85 to 0.96; the OP2 sample presented the lowest value (0.855; *p* < 0.05). Both the control and OP2 crumb presented values greater than 0.95 and were similar (*p* > 0.05). The pH of the samples was significantly different (*p* < 0.05); the lowest pH and, consequently, the highest TA were found for sample OP2, with values of 4.43 and 0.85%, followed by those for sample OP1, which had a pH value equal to 4.73 and a TA of 0.72%. The control had the highest pH and the lowest TA (6.56 and 0.021%). The specific volumes of the bread were similar (*p* > 0.05), with values between 2.83 and 2.96, which correspond to a desirable, good-quality bread in terms of its shape and size [49]. Finally, the bread width–height ratio did not significantly differ (*p* > 0.05), and values of 2.02, 2.04, and 2.00 were obtained for the control, OP1, and OP2 samples, respectively.

**Figure 4:**
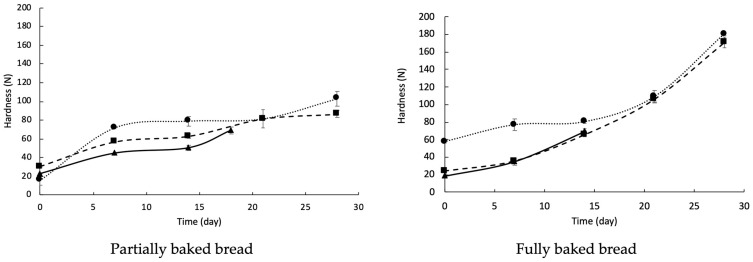
Hardness of partially baked bread during refrigerated storage (4 °C) and of fully baked bread after storage. Control (▲), bread supplemented with optimized mixture 1 (•), and bread supplemented with optimized mixture 2 (■).

Figure 4 shows the bread’s hardness during storage, which increased over time. This behavior has been widely described by other authors and is primarily attributed to the staling bread phenomenon [15,28,47,49]. The hardness in the control bread increased rapidly for seven days, then decreased until 14 days of storage. Subsequently, the bread presented a new increase in hardness until 18 days of storage, when there was evident mold growth on the surface.

Likewise, the samples to which sourdough had been added showed a very rapid increase in hardness for 7 days, and the bread from optimized mixture 1 reached an asymptotic value from 7 to 28 days of storage. The bread hardness with optimized mixture 2 was decreased after 7 days and subsequently increased slightly after 21 days of storage, to then remain steady. Similar hardness trends have been reported by other authors when analyzing the effect of the addition of sourdough fermented with LAB to bread formulations [15,47,50,51]. For instance, Zhao et al. [47] evaluated the addition of wheat germ fermented with *Lpb. plantarum* dy-1 into a bread formulation and observed an increase in the bread’s hardness at the beginning of storage. Later, the bread presented an asymptotic period and, again, a slight rise in hardness. The OP1 bread had the greatest hardness, which was related to the lower moisture content. Previous reports have shown that moisture has an inverse relationship with hardness; the lower the moisture, the harder the bread tends to be [27,49].

Figure 4 shows the hardness of the fully baked breads. In general, the hardness of the fully baked bread increased significantly (*p* < 0.05) during the storage compared with that of the partially baked counterpart, largely due to the staling process of the bread. OP1 bread presented greater hardness after 14 days of storage. After 21 days, the hardness of the three samples was similar, which could be attributed to staling and crust formation during the final baking. The fully baked breads from the control and OP2 samples had similar hardness values.

### 3.4. Bread Sensory Analysis

The sensory analysis of the loaves of bread is shown in Table 8. The control received higher scores for all the attributes; however, for odor, color, crumb appearance, crust appearance, flavor, and general acceptability, the three breads were not significantly different (*p* > 0.05). Only the texture was significantly different (*p* < 0.05) between the breads; the control had the highest score (7.58), followed by the OP2 sample (6.78) and, finally, by the OP1 sample (6.68). These texture scores agree with previous findings on hardness, which was discussed in Section 3.3. The general acceptability was slightly higher for the control (7.50), followed by the OP1 sample (7.10) and, finally, for the OP2 sample (6.78); however, the samples were not significantly different (*p* > 0.05). The sensory analysis demonstrates that the optimization was adequate for developing a sourdough bread with a similar sensory profile to a nonsupplemented one.

### 3.5. Antifungal Activity of Sourdough Fermented with Optimized Mixtures

Numerous studies have reported the antifungal activity of sourdough is primarily related with lactic acid and acetic acid [8,46,52]. Furthermore, using sourdough directly in dough formulations makes it easier to incorporate it as an ingredient.

**Figure 5:**
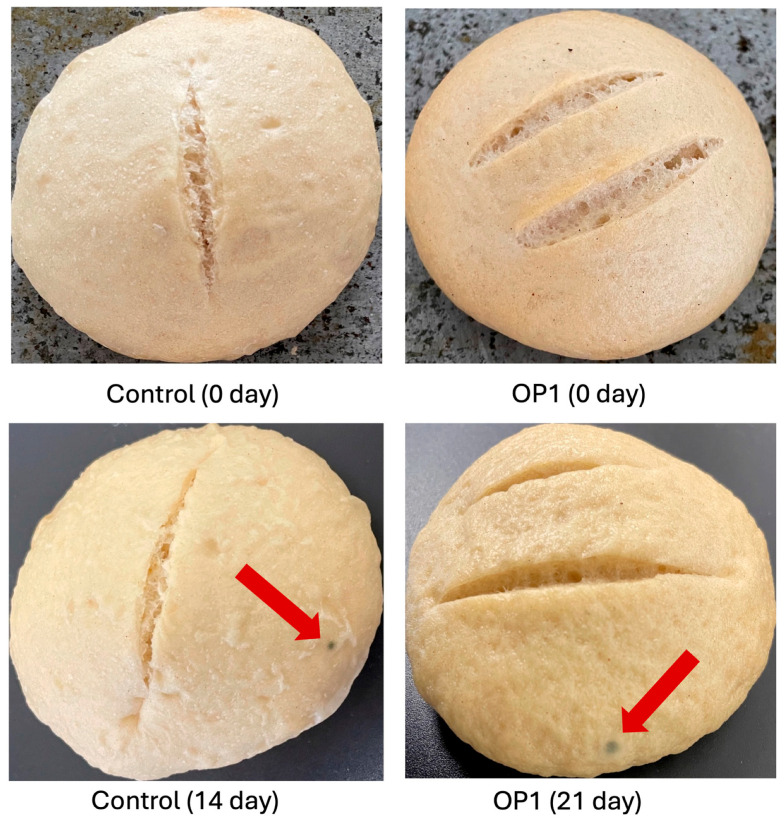
Fungal growth on the control loaves of bread, and bread to which optimized mixture 1 (OP1) was added during storage at 4 °C. Red arrows show fungal growth on the surface of the bread.

Table 9 shows the mold growth on the bread loaves during refrigerated storage at 4 °C. After 14 days of storage, 50% of the control bread showed evident mold growth on the crust of the PBB (Figure 5); at day 21, 100% of the control bread showed mold growth on the surface. These results agree with those obtained by Lainez et al. [27], who observed that PBB without antimicrobials showed fungal growth on the surface after 14 days of storage at 7 °C. For the OP1 bread, containing sourdough, only 30% of the bread had mold growth on the surface after 28 days of storage. In the case of OP2 bread, no mold growth was observed on the surface during the 28 days of storage. Despite the OP2 bread not showing fungal growth during storage, it was already hard (undesirable texture) and, thus, had lost quality.

## 4. Conclusions

A simplex-lattice mixture design was used to optimize the proportions of *Lpb. plantarum*, *Lcb. casei*, and *L. acidophilus* in sourdough, which was found to have practical benefits for the resulting bread quality and stability. The aqueous extracts from the sourdough fermented with the three different LAB mixtures demonstrated effective antifungal activity, associated with the production of organic acids. Mixtures with a higher proportion of *Lpb. plantarum* showed stronger inhibitory effects, producing the highest acetic acid concentration and extending the lag time for mold growth. The optimized LAB mixtures improved the physicochemical characteristics and extended the shelf-life of PBB during refrigerated storage by inhibiting mold growth. The PBBs that were fully baked and then that underwent sensory evaluation were found to have an overall acceptability similar to that of the control bread (without sourdough), being nonstatistically different (*p* > 0.05). The sensory analysis results showed that the optimization successfully allowed the formulation of a sourdough bread that closely resembled a nonsupplemented one in terms of sensory profile. The determined LAB proportions for sourdough fermentation, optimized through a mixture design, can effectively enhance sourdough bread’s antifungal properties and quality, providing a promising approach for extending bread shelf-life while maintaining desirable sensory attributes. These results highlight the significance of carefully choosing the appropriate lactic acid bacteria (LAB), determining their proportions to achieve specific objectives for successful sourdough fermentation, and developing bakery products.

## Figures and Tables

**Table 1:** Proportions of lactic acid bacteria for sourdough preparation in a simplex-lattice mixture design (X_1_ = *Lactiplantibacillus plantarum*; X_2_ = *Lacticaseibacillus casei*; and X_3_ = *L. acidophilus*).

Mixture (M)	Lactic Acid Bacteria Proportions (Fractions)		
+	*X*_1_	*X*_2_	*X*_3_
1	1	0	0
2	0.167	0.667	0.167
3	0	1	0
4	0.667	0.167	0.167
5	0.333	0.333	0.333
6	0.167	0.167	0.667
7	0	0	1
8	0.5	0	0.5
9	0	0.5	0.5
10	0.5	0.5	0

**Table 2:** Physicochemical properties of the aqueous extracts, mold growth rate, and lag phase for each analyzed mixture of lactic acid bacteria.

					*P. chrysogenum*		*P. corylophilum*	
Mixture	pH	TA (%)	mM Lactic Acid	mM Acetic Acid	μ (1/h)	Lag (h)	μ (1/h)	Lag (h)
1	3.06 ± 0.03 f	0.60 ± 0.01 a	120.76 ± 1.41 d	568.53 ± 6.29 a	0.13 ± 0.01 b	78.52 ± 5.80 a	0.14 ± 0.04 a	85.20 ± 11.88 a
2	3.30 ± 0.01 d	0.20 ± 0.03 bc	324.41 ± 0.86 a	331.46 ± 2.08 b	0.20 ± 0.01 ab	69.27 ± 0.60 ab	0.20 ± 0.01 a	67.32 ± 4.39 ab
3	3.24 ± 0.02 e	0.19 ± 0.01 bcd	333.41 ± 6.07 a	194.03 ± 3.74 c	0.23 ± 0.05 a	71.52 ± 2.26 ab	0.22 ± 0.04 a	65.15 ± 5.88 ab
4	3.32 ± 0.01 cd	0.17 ± 0.01 bcd	222.95 ± 12.13 c	436.22 ± 18.3 b	0.18 ± 0.01 ab	60.27 ± 5.43 ab	0.18 ± 0.01 a	62.70 ± 4.97 ab
5	3.36 ± 0.02 b	0.19 ± 0.03 bcd	276.59 ± 2.61 b	6.73 ± 5.32 d	0.17 ± 0.02 ab	59.99 ± 1.32 ab	0.18 ± 0.02 a	58.63 ± 15.25 ab
6	3.35 ± 0.01 bc	0.12 ± 0.02 cd	235.06 ± 0.15 c	3.01 ± 0.07 d	0.18 ± 0.02 ab	52.24 ± 2.68 b	0.18 ± 0.01 a	54.52 ± 5.56 ab
7	3.57 ± 0.01 a	0.11 ± 0.01 d	222.1 ± 16.47 c	2.95 ± 0.01 d	0.16 ± 0.04 ab	59.58 ± 16.77 ab	0.17 ± 0.04 a	69.87 ± 13.23 ab
8	3.36 ± 0.01 b	0.12 ± 0.01 cd	142.67 ± 2.04 d	350.17 ± 9.25 b	0.17 ± 0.04 ab	59.26 ± 5.67 ab	0.17 ± 0.03 a	65.88 ± 5.38 ab
9	3.04 ± 0.01 f	0.20 ± 0.02 bcd	341.79 ± 4.18 a	76.92 ± 0.49 d	0.17 ± 0.04 ab	65.62 ± 5.67 ab	0.17 ± 0.03 a	46.46 ± 5.38 b
10	3.06 ± 0.01 f	0.23 ± 0.01 b	238.52 ± 1.47 c	17.81 ± 20.32 d	0.21 ± 0.01 ab	66.78 ± 0.01 ab	0.22 ± 0.01 a	67.98 ± 0.82 ab

TA (%) is the titratable acidity expressed as lactic acid. For each column, different lowercase letters indicate a significant difference (*p* < 0.05).

**Table 3:** Coefficients of the polynomial models for the different studied responses analyzed through the mixture design of the three lactic acid bacteria: *Lactiplantibacillus plantarum* (LP), *Lacticaseibacillus casei* (LC), *Lactobacillus acidophilus* (LA).

Term *	pH	TA	mM Lactic Acid	mM Acetic Acid	Log R	*Penicillium chrysogenum*		*Penicillium corylophilum*	
						μ (1/h)	Lag (h)	μ (1/h)	Lag (h)
LP	4.009	0.073	127.7	593.0	−0.834	0.132	77.77	0.1425	83.04
LC	3.261	0.212	330.6	248.8	−0.026	0.233	73.30	0.2204	67.84
LA	3.609	0.109	213.7	−44.7	2.142	0.1594	58.48	0.1700	68.83
LP * LC	−1.066	0.468	116.5	−1247	6.270	0.108	−37.10	0.1390	−22.20
LP * LA	−1.844	0.101	−55.4	260.0	−2.940	0.089	−49.00	0.0797	−47.50
LA	−1.020	0.093	296.2	−23.0	−0.800	−0.124	−4.60	−0.1160	−75.40
Correlation coefficient (r)	0.924	0.948	0.977	0.868	0.823	0.828	0.790	0.786	0.782
Lack-of-fit (*p*-value)	0.110	0.087	0.098	0.105	0.107	0.201	0.505	0.435	0.435

* significant (*p* < 0.05).

**Table 4:** Optimized response values and weights used to obtain two mixtures of lactic acid bacteria according to the simplex-lattice mixture design (SLMD).

Optimization 1			Optimization 2		
	Goal	Weight		Goal	Weight
Log R	Target (0.65)	0.50	Log R	Target (0.65)	0.50
Lag PCH	Maximum	0.25	Lag PCH	Maximum	0.15
Lag PC	Maximum	0.25	Lag PC	Maximum	0.15
			Acetic acid (mM)	Target (160)	0.20

**Table 5:** Optimal lactic acid bacteria (LAB) mixture composition.

	LAB Proportion	
LAB	Optimization 1	Optimization 2
*Lpb. plantarum*	0.67	0.34
*Lcb. casei*	0.29	0.29
*L. acidophilus*	0.04	0.37

**Table 6:** Quality properties and physicochemical characteristics of partially baked bread during refrigerated storage (4 ± 1 °C).

Control						
Time (Days)	Specific Volume (cm^3^/g)	W/H Ratio	pH	TA (%)	a_w_	Moisture (%wb)
0	3.01 ± 0.16 a	2.14 ± 0.08 a	6.54 ± 0.05 Ac	0.02 ± 0.01 Ac	0.93 ± 0.005 Aa	40.68 ± 1.99 Aa
7	ND	ND	6.21 ± 0.03 Bc	0.03 ± 0.01 Ac	ND	ND
14	ND	ND	5.82 ± 0.01 Cc	0.03 ± 0.01 Ac	ND	ND
18	ND	ND	5.83 ± 0.02 Cc	0.04 ± 0.00 Ac	0.95 ± 0.002 Aa	40.07 ± 0.95 Aa
**Optimization 1**						
**Time (Days)**	**Specific Volume (cm^3^/g)**	**W/H Ratio**	**pH**	**TA (%)**	**a_w_**	**Moisture (%wb)**
0	2.88 ± 0.17 a	2.13 ± 0.02 a	4.80 ± 0.09 Ab	0.55 ± 0.01 Bb	0.93 ± 0.001 Aa	33.93 ± 1.01 Ab
7	ND	ND	4.84 ± 0.06 Ab	0.50 ± 0.01 Bb	ND	ND
14	ND	ND	4.83 ± 0.03 Ab	0.61 ± 0.01 Ab	ND	ND
21	ND	ND	4.47 ± 0.03 Bb	0.71 ± 0.01 Ab	ND	ND
28	ND	ND	4.49 ± 0.02 Bb	0.70 ± 0.01 Ab	0.94 ± 0.01 Aa	33.50 ± 1.28 Ab
**Optimization 2**						
**Time (Days)**	**Specific Volume (cm^3^/g)**	**W/H Ratio**	**pH**	**TA (%)**	**a_w_**	**Moisture (%wb)**
0	2.80 ± 0.13 a	2.01 ± 0.01 a	4.38 ± 0.02 Aa	0.80 ± 0.00 Ba	0.93 ± 0.002 Aa	37.66 ± 1.75 Aab
7	ND	ND	4.37 ± 0.01 Aa	0.82 ± 0.00 Ba	ND	ND
14	ND	ND	4.33 ± 0.01 Aa	0.86 ± 0.01 Ba	ND	ND
21	ND	ND	4.34 ± 0.04 Aa	0.86 ± 0.01 Aa	ND	ND
28	ND	ND	4.19 ± 0.01 Ba	0.95 ± 0.01 Ba	0.95 ± 0.003 Aa	37.67 ± 1.44 Aa

Different capital letters indicate a significant difference (*p* < 0.05) between samples at different storage times. Lowercase letters indicate a significant difference (*p* < 0.05) among types of bread. ND—not determined.

**Table 7:** Quality parameters and physicochemical characteristics of fully baked breads.

	Control		Optimization 1		Optimization 2	
	Crust	Crumb	Crust	Crumb	Crust	Crumb
Moisture (%wb)	28.10 ± 1.53 A	46.52 ± 1.55 A	20.58 ± 1.49 B	40.32 ± 0.13 C	26.76 ± 1.36 C	42.07 ± 0.12 B
a_w_	0.840 ± 0.002 A	0.958 ± 0.003 AB	0.853 ± 0.003 A	0.855 ± 0.001 B	0.851 ± 0.002 A	0.960 ± 0.001 A
pH	6.56 ± 0.05 A		4.73 ± 0.11 B		4.43 ± 0.05 C	
TA (%)	0.021 ± 0.01 C		0.72 ± 0.01 B		0.85 ± 0.01 A	
Specific volume (cm^3^/g)	2.96 ± 0.20 A		2.83 ± 0.16 A		2.96 ± 0.08 A	
W/H ratio	2.02 ± 0.06 A		2.40 ± 0.40 A		2.00 ± 0.03 A	

Different letters indicate a significant difference (*p* < 0.05) among types of bread (rows).

**Table 8:** Scores of the sensory analysis for loaves of bread with untrained judges.

	Control	Optimization 1	Optimization 2
Odor	7.10 ± 1.77 a	7.18 ± 1.55 a	7.33 ± 1.73 a
Color	7.60 ± 1.41 a	7.85 ± 1.48 a	7.45 ± 1.48 a
Crumb appearance	7.50 ± 1.41 a	6.93 ± 1.77 a	6.85 ± 1.64 a
Crust appearance	7.68 ± 1.37 a	7.40 ± 1.78 a	7.33 ± 1.65 a
Flavor	7.10 ± 1.98 a	7.03 ± 1.91 a	6.35 ± 1.90 a
Bread texture	7.58 ± 1.48 a	6.68 ± 1.64 b	6.78 ± 1.61 ab
General acceptability	7.50 ± 1.28 a	7.10 ± 1.43 a	6.78 ± 1.42 a

For each attribute, different letters indicate a significant difference (*p* < 0.05) among types of bread.

**Table 9:** Mold growth (%) on partially baked bread during refrigerated storage (4 °C).

Time (Day)	Control (%)	Optimization 1 (%)	Optimization 2 (%)
0	0	0	0
4	0	0	0
7	0	0	0
14	50	0	0
21	100	10	0
28	100	30	0

## Data Availability

The original contributions presented in the study are included in the article, further inquiries can be directed to the corresponding author.
